# Revisiting Plant Heterosis—From Field Scale to Molecules

**DOI:** 10.3390/genes12111688

**Published:** 2021-10-24

**Authors:** Attiq ur Rehman, Trang Dang, Shanzay Qamar, Amina Ilyas, Reemana Fatema, Madan Kafle, Zawar Hussain, Sara Masood, Shehyar Iqbal, Khurram Shahzad

**Affiliations:** 1 Horticulture Technologies, Production Systems Unit, Natural Resources Institute (Luke), Toivonlinnantie 518, 21500 Piikkiö, Finland; attiq.rehman@luke.fi; 2 Department of Agricultural Sciences, Faculty of Agriculture and Forestry, The University of Helsinki, 00790 Helsinki, Finland; kaflemadan10@gmail.com; 3 Institute of Integrative Biology, ETH Zürich, 8092 Zürich, Switzerland; 4 Department of Agricultural Biotechnology, National Institute of Biotechnology and Genetic Engineering, Pakistan Institute of Engineering and Applied Science, Faisalabad 38000, Pakistan; shanzayq3@gmail.com; 5 Department of Botany, Government College University, Lahore 54000, Pakistan; aminailyas1510@gmail.com; 6 Department of Plant Breeding, Swedish University of Agricultural Sciences (SLU), SE-230 53 Alnarp, Sweden; fatemareemana@gmail.com; 7 Department of Seed Science and Technology, Ege University, Bornova, Izmir 35100, Turkey; 8 Environmental and Plant Biology Department, Ohio University, Athens, OH 45701, USA; zh729420@ohio.edu; 9 University Institute of Diet and Nutritional Sciences (UIDNS), Faculty of Allied Health Sciences, University of Lahore, Lahore 54000, Pakistan; sarah.13494@gmail.com; 10 IMPLANTEUS Graduate School, Avignon Université, 84000 Avignon, France; iqbalshehryar781@gmail.com; 11 Department of Plant Breeding and Genetics, The University of Haripur, Haripur 22620, Pakistan; KShahzad@uoh.edu.pk

**Keywords:** heterosis, hybrid vigor, inbreeding depression, genetic models, molecular basis, crop plants

## Abstract

Heterosis refers to the increase in biomass, stature, fertility, and other characters that impart superior performance to the F1 progeny over genetically diverged parents. The manifestation of heterosis brought an economic revolution to the agricultural production and seed sector in the last few decades. Initially, the idea was exploited in cross-pollinated plants, but eventually acquired serious attention in self-pollinated crops as well. Regardless of harvesting the benefits of heterosis, a century-long discussion is continued to understand the underlying basis of this phenomenon. The massive increase in knowledge of various fields of science such as genetics, epigenetics, genomics, proteomics, and metabolomics persistently provide new insights to understand the reasons for the expression of hybrid vigor. In this review, we have gathered information ranging from classical genetic studies, field experiments to various high-throughput omics and computational modelling studies in order to understand the underlying basis of heterosis. The modern-day science has worked significantly to pull off our understanding of heterosis yet leaving open questions that requires further research and experimentation. Answering these questions would possibly equip today’s plant breeders with efficient tools and accurate choices to breed crops for a sustainable future.

## 1. Introduction

Heterosis, alternatively known as ‘outbreeding enhancement’, is characterized by the increase in vigor, biomass, speed of development and fertility relative to the average of 

The genetically diverged two parents [1]. Throughout history, heterotic phenomenon was perceived in different terms in different civilizations, but only by the 1870s, the term “heterosis” was fully described when Charles Darwin observed increased fertility, height and overall weight in cross-pollinated species compared to their self-pollinated counterparts [2]. The development of hybrid crops is undeniably one of the landmark innovations in the global seed sector that harvested the heterosis and resulted in a significant increase in crop yields and the respective revenue earned from crop husbandry per se. For example, China has increased its rice production by 44.1% on account of hybrid rice [3], and the European market has been giving great preferences to hybridsfor many of its main crops such as sugar beet, rapeseed and rye [4]. 

Nevertheless, not all parental combinations in a cross breeding program results in the superiority of hybrid progenies, suggesting that only particular combinations of parents would play a central role in the expression of heterotic effect [5]. In fact, heterosis is frequently encountered in allogamous plants that are prone to inbreeding depression, rather than autogamous plants that prefer selfing [6]. This phenomenon could be explained via genetic distance, discussed systematically the first time by East in year 1936, and since then it has been among the most popular research topics in plant breeding [7]. According to East, the extent of the heterotic effect is proportional to the genetic distance between the two parental lines, but this relationship ceases at an optimum level and declines beyond that point due to either reproductive barriers or lack of adaptation of the parents. East’s discovery was bolstered by Chen [8], who collected and analysed the data from twelve independent research studies on *Arabidopsis thaliana* and *Zea Mays* to show that hybrids of these plant species derived from distanced parents expressed significant vigour in terms of biomass and fitness as long as their parents were still within the limit of reproductive barriers [8]. 

**Figure 1:**
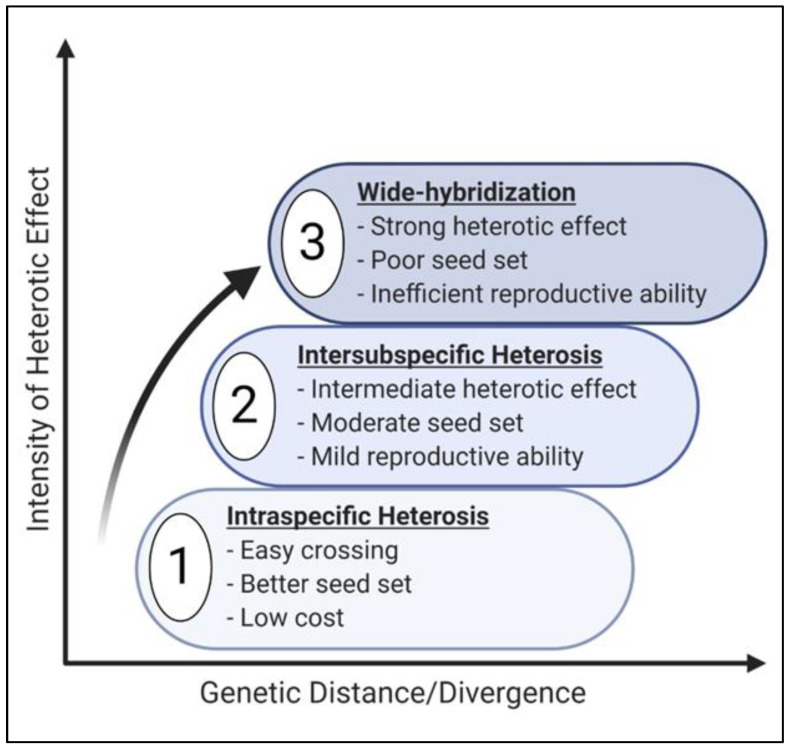
Classification of heterosis in plant breeding based on genetic distance and utilization.

From the perspectives of plant breeding, heterosis is categorized into three groups based on the parental genetic distance i.e. (Figure 1): (i) *intraspecific heterosis*: involving crosses from two accessions which are members of the same species; (ii) *inter-subspecific heterosis*: a result of hybridization between two subspecies which has evidently been exploited in hybrid rice [9,10]; and (iii) *wide hybridization*: which is the consequence of crossing between two individuals belonging to different species from distant gene pools and are directly aimed at boosting up plant biomass [11,12]. Wide hybridization events give rise to the development of novel allopolyploid species such as *Triticum aestivum* L. (approximately 9000 year ago) [13] and *Fragaria × ananassa* (approximately 300 years ago) [14].

Heterosis in plant breeding is often described with different terms andextended definitions. Alternative terminologies such as ‘Heterobeltiosis’ and ‘Commercial Heterosis’ are used to indicate superior performance of a hybrid compared to either better-performing parents or a control cultivar, respectively. These definitions might serve as a useful measure for the development of a crop variety but are not necessarily related to the population improvement on the genetic scale. In addition, the pronounced phenotypic expression in the progenies in relation to their parents can be considered either positive or negative depending on the breeding objectives. For instance, positive heterosis for days to flowering is alternative to negative heterosis for the rate of plant development, because a ‘late’ flowering plant would have positive value for days to flowering but will have less positive value for the rate of development as it may mature at a slower pace. Therefore, the perception of heterosis can be a simple artifact of the researcher’s choice in terms of the phenotypic measure/trait under investigation [15]. Moreover, the occurrence of heterosis can be discerned as a ‘system-wide’ phenomenon that results in enhanced size, vigor, resistance to pest/disease, or climatic factors influencing crop performance on a crop and is taken as an overall ‘effect’. This standpoint of heterosis has not only made plant breeders benefit from this phenomenon to breed better crops but has also given rise to the search for a unifying theory and investigations on various scientific levels to understand the underpinnings of hybrid vigor. 

In summary, heterosis in plants has been a hot topic for plant science researchers worldwide for a long time. Therefore, in this article, we systematically revise the underlying mechanisms of heterosis in plants with a particular reference to plant breeding, thereby suggesting the orientations for future research and the manifestation of hybrid vigor.

## 2. Understanding the Molecular Basis of Heterosis

There are various ways to study heterosis on the molecular scale, for instance; genome organization studies, transcriptome-wide gene expression profiling, and studying allele-specific contributions to gene expression [4]. Many of these molecular approaches eventually root down to the most basic and widely acceptable models of heterosis: combined allelic expression and diverse allelic interactions in a hybrid [16]. However, heterosis remains an intriguing research subject until now, and thus, additional knowledge to understand this phenomenon has been and is continuously uncovered, thanks to state of the art research techniques in epigenetics, genomics, proteomics, and metabolomics. Here we summarize and comprehend the current perspectives explaining the existence of hybrid vigor in plant species.

### 2.1. Genetic Models Explaining the Phenomenon of Heterosis

**Figure 2:**
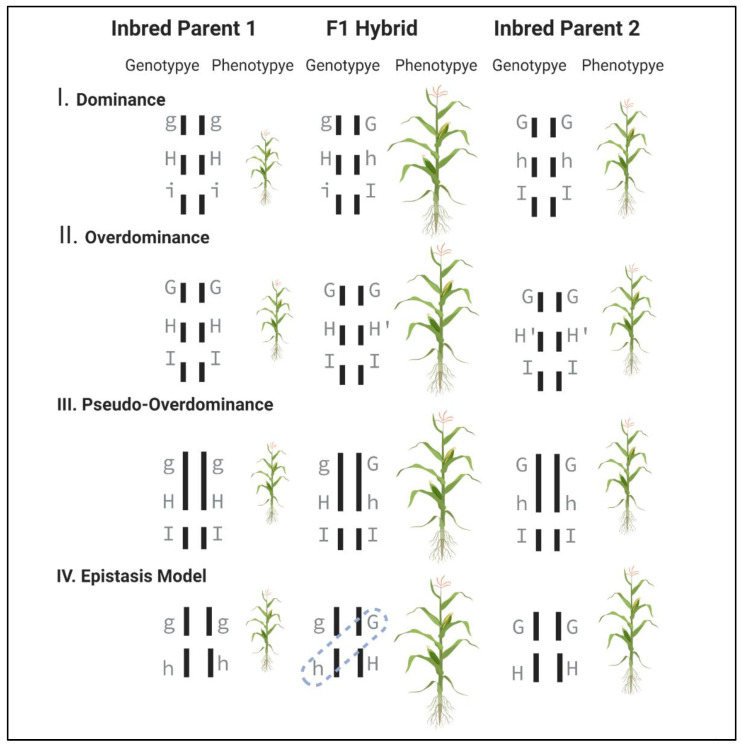
Genetic models for heterosis. Diagrams represent that the hypothetical phenotype or trait is influenced by multiple linked or unlinked loci (e.g., ‘g’, ‘h’, ‘i’). (I) **Dominance Model**: Inbred parents 1 and 2 exhibit marginally deleterious alleles in homozygous form (g and i in parent 1; h in parent 2). In F1 hybrid, complementation of superior alleles (G, H, I) occur at each locus resulting in a superior F1 phenotype. (II) **Overdominance Model**: Homozygous alleles at locus ‘h’ are different for both the inbred parents (HH and H’H’). In F1 hybrid, the interaction H and H’ produces a superior phenotype in comparison to both homozygous parents. (III) **Pseudo-Overdominance Model**: The superior performance of F1 hybrid is due to a small chromosomal region harboring two or more loci (e.g., g and h) linked in repulsion, in which the complementation of G and H is mimicking overdominance. (IV) **Epistasis Model**: The superior performance of F1 hybrid is due to the interaction between two different loci.

Genetic models are predominantly popular and are considered as a prerequisite approach to understand the rational aspects of heterosis. Different models have been evolved over time to explain the occurrence of heterosis, but none of them is able to completely explain the entire basis of this phenomenon alone. The three most important models are Dominance, Over-dominance and Pseudo-overdominance model (Figure 2). In addition, Epistasis has also been under discussion as an underlying reason for crop heterosis (Figure 2, IV). 

#### 2.1.1. Dominance Model

The hypothesis regarding the “Dominance” model focuses on the argument that heterosis is the outcome of complementation of recessive alleles present in inbred parents [17]. Inbred parents exhibit homozygous alleles with deleterious effects (inbreeding depression), the effects of which are masked in a hybrid combination because the superior alleles will complement the effects of inferior ones. These complementations happen at multiple loci, leading to the non-expression of the deleterious effects (caused by recessive alleles), resulting in a better performing F1 hybrid (Figure 2, I). Consequently, natural selection reduces the deleterious alleles or encourages their tight linkage with the beneficial alleles [18]. This way, the model assumes that heterozygosity is not a major requirement of heterosis instead the increased number of superior loci is the main contributor [2]. The possible gap in the Dominance model is that it is unsure whether all the recessive allele complementations would result in an additive effect on the final phenotype [19].

#### 2.1.2. Overdominance Model

Heterosis can not be described solely based on the complementation of deleterious alleles, which led to the development of another hypothesis in classical genetics called the “Overdominance” model. This model proclaims that heterosis is merely thesuperiority of the heterozygotes over each of the homozygotes [20]. The interactions among diverse alleles in heterozygous genotypes, which occur in neither of the homozygous states (dominant/recessive), give rise to superior trait performance (Figure 2, II). The model is supported by the fact that heterozygosity for small genomic regions usually causes a heterotic response. 

#### 2.1.3. Pseudo-Overdominance Model

The imbalance between the Dominance and Overdominance models resulted in the advocacy of the “Pseudo-overdominance” model. This model is based on the fact that some small genomic regions in hybrids could have variations in repulsion at two or more different genes. Those variations complement and result in superior phenotypes, which apparently looks like an Overdominance action [21]. This model demonstrates that homozygous dominant (favourable) alleles are linked with recessive (unfavourable) alleles in parental lines, but after the hybridization, they attain a heterozygous state and behave as an Overdominant locus (Figure 2, III). 

In summary, the major challenge to analyse the basis of heterosis based on all these models is how to discover the possible roles of multiple genes contributing to the superior performance of F1 hybrids.

#### 2.1.4. Epistatic Model

It is important to recall that neither the dominance nor the overdominance models can fully elucidate the phenomena of heterosis. At times, epistatic interactions are also taken into consideration while discussing the principles of heterosis. Epistasis is defined as the interaction of genes from at least two loci that affects the phenotypic expression of a characteristic (Figure 2, IV). The study by Powers suggested that both intra-allelic and inter-allelic interactions, as well as crosstalk between genes and the environment seem to be implicated in the phenomenon of heterosis [22]. Particularly in scenarios with no dominance or even partial dominance in certain genes that are not receptive to an improvement in the quantitative character, the heterotic expression is still exhibited. For instance, a study by Liang [23] on determining the genetic basis of heterosis in Upland cotton (*Gossypium hirsutum* L.) has shown substantial boost in hybrid productivity for boll number per plant, directly contributing to lint yield as a result of epistatic interaction [23,24]. Furthermore, to understand the phenomena of heterosis at the metabolic level, metabolite profiling was done on two mapping populations of Arabidopsis (369 RILs and their testcross offspring, and 41 introgression lines (ILs) and their test crosses, respectively) by Lisec et al. [25]. In the first population, the researchers discovered 147 QTLs for metabolite absolute mid-parent heterosis (aMPH), as well as 153 and 83 QTL for enhanced additive and dominant effects, respectively. In conclusion, Epistasis was found as a significant contributor to metabolite heterosis in *Arabidopsis*. 

### 2.2. Genomic View of Heterosis

The four genetic models based on classical genetics explain the hybrid vigour for diploid genomes quite reasonably, hence are popular among plant breeders [16]. However, from a broader perspective, heterosis should be considered as a genome-wide phenomenon reflecting global changes at both expression levels of genes and proteins. It might be a consequence of genomic differences among parental lines such as genomic structure, presence and distribution of specific genes in the crossed individuals that generate a net positive effect [26]. Such changes in hybrid plants, whether additive or non-additive, possibly affect the regulatory and metabolic pathways associated with plant traits [27]. Logically, such a combination of differential gene expression due to genome incorporation, which affects a major regulatory pathway, could determine the manifestation of heterosis.

Studies on maize hybrids have revealed that the genetic distance between the parental inbred lines is correlated to the overall heterotic effect. These results provide the basis for investigating the impact of genomic constitution of inbred/parental lines onhybrid vigor. Later, it has been demonstrated that approximately 10% of the genes present in any of the maize inbred parents (B73 and Mp17) are generally not found in another genotype. Hence, the missing genes in one parent are complemented by the other in a hybrid offspring [2]. 

Moreover, QTL analyses have revealed fewer loci with significant effects contributing to the phenomenon of heterosis, but no single associated QTL has been cloned to date. Although due to the current advancements in high-throughput technologies, this could possibly be achieved in the coming days [28]. Several studies have reported genes that express potential roles in heterosis among various plant species. These genes are found associated with the expression of higher heterotic effect when overexpressed, silenced, mutated or epigenetically modified (Table 1).

### 2.3. Epigenetic View of Heterosis

**Figure 3:**
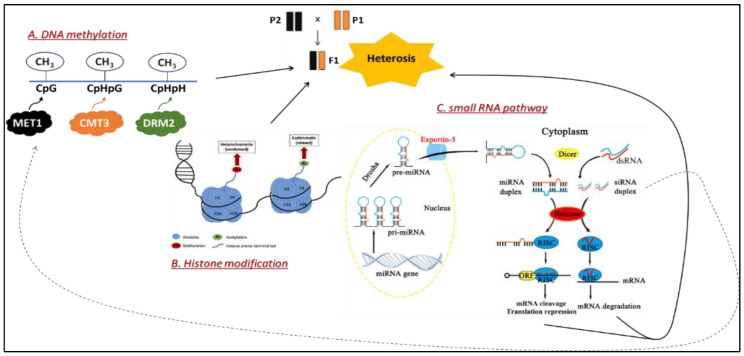
Summary of different epigenetic models that possibly associate with heterotic effect. (**A**) DNA methylation happens when a methyl group (CH3-) binds to the 5′ end of cytosine base (C), usually due to the activity of METHYTRANSFERASE 1 (MET1), CHROMOMETHYLASE 3 (CMT3) and DOMAINS REARRANGED METHYLATION 2 (DRM2). These enzymes induce different DNA methylation models. The appearance of MET1 and CMT3 would lead to symmetric methylation (CG, CHG), while DRM2 would be responsible for an asymmetric model (CHH), in which H can be A, T, or C [31,32]. (**B**) Histone modification refers to the changes in histone proteins that would significantly affect their associated DNA regions, modifying the transcriptional capability of the genes on those regions. The alteration of histone proteins can be due to the addition of chemical groups both on histones’ globular domain and at the N-terminal tails. Those chemical groups are called histone marks. The most well-studied histone marks include Acetylation (Ac) and methylation (Me) [33,34]. (**C**) sRNA plays both direct and indirect roles in regulating gene expression. The direct role is to activate RNA Induced Silencing Complex (RISC) that would silence targeted genes after transcription. This activity involves either microRNAs (miRNAs) or small interfering RNAs (siRNA). MicroRNAs (miRNAs) are produced endogenously from the transcription of MIR genes, while siRNA synthesis is mostly stimulated by the presence of abnormal double-stranded RNAs produced from transposons in heterochromatic regions or by invading viral RNAs. These two types of sRNA can be cleaved by DICER LIKE 1,2,3 or 4 in the cytoplasm into short sequences of 20–27 nucleotides, which would either lead to activation of the RISC to mediate post-transcriptional gene silencing (direct gene regulation) or to initiate the DNA methylation (indirect gene regulation) [35,36].

In addition to the genetic basis, some hypotheses also suggest possible contributions of non-genetic factors underlying heterosissuch as epigenetics. Epigenetics refers to the study of heritable changes in gene expression that do not involve the genotype of crop plants but the alterations in chromatin architecture and/or the post-transciptional process. The mechanisms that direct plants to translate their genotype in different directions lie in DNA methylation, histone modification and small RNA (sRNA) pathways (Figure 3).

#### 2.3.1. Heterosis and DNA Methylation

Numerous studies have suggested the association between DNA methylation and heterotic effects in hybrid plants. For example, one study proved that a great majority of cytosine methylation sites in maize parental lines had changed in their hybrids, suggesting the possible relevance of methylation-pattern remodeling to heterosis [4]. This hypothesis was further examined by later experiments on different model plants. He et al. [37] observed a strong correlation between cytosine methylation (mC) patterns and genetic expression changes among both rice hybrids and their inbred parental lines. Two years later, two independent studies by Shen et al. [38] and Greaves et al. [39] performed genome-wide methylation profiling of *Arabidopsis thaliana* parental inbred lines and their reciprocal hybrid lines that displayed heterosis for biomass. They both discovered that F1 hybrids showed higher overall levels of DNA methylation compared to their parents. These discoveries suggest a possible role of DNA methylation in the expression of hybrid vigor. Recently, Lauss et al. [40] crossed Arabidopsis inbred parents with different specifically induced methylated regions, producing a large number of heterotic hybrids with diverse epigenetic patterns (epiHybrids). The specific methylated regions in the parental genomes were statistically analyzed with hybrid performance, revealing strong correlations and thus, supporting the hypothesis of direct or indirect influence of epigenetics in parental lines on hybrid heterotic performance. However, the specific methylated regions and their respective mechanism(s) to improve hybrid performance from their inbred parents remained unclear and became an interesting topic for scientific research

#### 2.3.2. Heterosis and Histone Modifications

**Figure 4:**
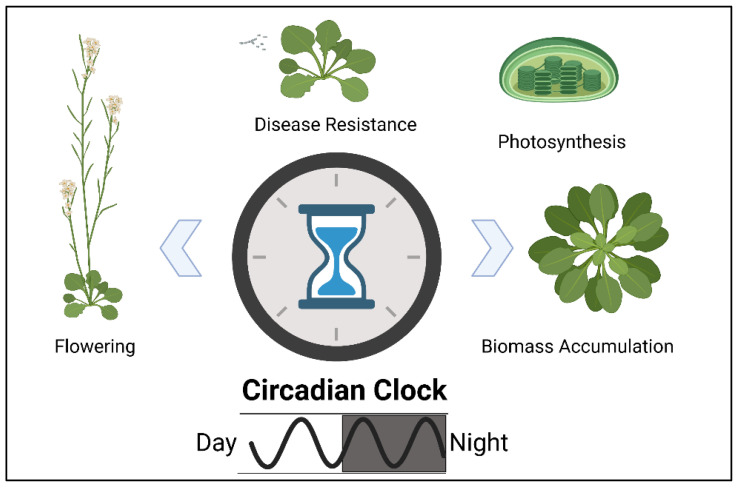
Various plant processes affected by Circadian rhythm defining overall fitness.

Histone modification affects many genes and flanking regions on the associated DNA molecules. Therefore, it is more challenging to study the relationship between histone modification and heterosis because of its complexity. The most noticeable attempts to uncover the possible role of histone modification on heterosis focus on the well-known model genome of *Arabidopsis thaliana*. In 2009, Ni and colleagues, by observing Arabidopsis F1 hybrids’ circadian clock and its involving genes, discovered that the transcription of these genes changed in association with histone modifications [41]. This finding is important because the circadian clock plays an essential role in many biological processes of plants, including starch biosynthesis and growth rate (Figure 4). Plants with internal circadian rhythm matching their living environments are more vigorous than plants that fail to keep this synchronization [42]. Therefore, the epigenetically histone-mediated transcriptional changes of genes involved in the circadian rhythmsmay be associated with the performance of F1 hybrids.

Together with the evidential support from Arabidopsis studies, further research on crop plants has also been conducted to discover the relation between histone modification and heterosis. Maize F1 hybrids showed significant expression variations in the key histone HTA112 on endosperm transcriptomes compared to their parental inbred lines [43]. The study provided an entry point to the investigation of specific histone modification regulating crop hybrid performance. In rice, three global histone marks patterns (H3K4me3, H3K9ac, and H3K27me3) were analyzed among two rice subspecies, ‘japonica’ and ‘indica’, and their F1 hybrids using high-throughput ChIP-Seq [37]. Consequently, H3K4me3 (transcriptional activation mark) and H3K27me3 (transcriptional repression mark) were expressed differently between hybrids and parents. These findings contribute to the demonstration of possible associations between alterations of epigenetic histone modifications and heterosis.

#### 2.3.3. Small RNA (sRNAs)—Role in Epigenetic Regulation and Heterosis

Small RNAs (sRNA) plays the role of transcriptional silencing, post-transcriptional silencing function and are involved in RNA-directed DNA methylation (RdDM) pathway (Figure 3). sRNAs of 24 nt are associated with transcriptional gene silencing by targeting DNA methylation to complementary sequences [44]. Many studies support the hypothesis that these multi-functioning sRNA might also be involved in heterosis. As expected, the amount of 24-nt siRNA accumulated in the Arabidopsis hybrids was significantly lower than that of Col and Ler parents, correlating to the decrease in CpHpH methylation patterns [45]. A similar finding was reported from an Arabidopsis genome-wide sRNA sequencing project by Shen and colleagues [38]. In addition, other crops such as wheat, maize and rice also underwent sRNA accumulation analysis and showed significant variation of sRNA levels between hybrids and parental lines in many independent studies [46,47,48].

Most of the studies up-to-date on possible links between epigenetics and heterosis have been based on statistical correlation models without a clear explanation of underlying mechanism(s). It would require a long journey to precisely unravel the contribution of epigenetics to heterosis, and thus, opens an interesting research domain for plant breeding science.

### 2.4. Heterosis, Proteomics and Transcriptomics

Several proteomic studies in relation to the phenomena of hybrid vigor in crops have been conducted in the last decade [49,50]. The information obtained provides global knowledge of protein variations and their impact on the hybrids compared to their parents. Formerly, intensive research had been conducted on understanding single-molecule models for heterosis. But presently, published investigations and data propose that heterosis is the outcome of variable gene expressions, the associated pathways and progressions that are known and yet undiscovered [50].

In the past decade, it has been revealed through experimentation that the proteome plays a vital role in the expression of heterosis, providing an improved stress response, higher photosynthetic and glycolysis rate, and better disease resistance [51]. However, genetic and other biochemical data are important to testify its precise role in the phenomena across various crop species [50]. In some conditions, hybrid vigor can be explicated by heterozygote advantage that expresses diverse protein isoforms encoded by the same locus [29,49]. Isozymes are defined as different variants of the same enzyme with identical functions that are present in the same individual [50,52] and are considered as one of the primitive proteomic tools to investigate heterosis. They can be used to identify genetic affinity between plants by utilizing the data related to isozyme variability and the variability of genes encoding for the isozymes. However, studies represent that the isozymes deliver inadequate significance and contributions in the estimation of hybrid vigor and performance [53]. Investigating complex proteins and proteomes using more specialized techniques such as two-dimensional electrophoresis and mass spectrometry would be required to analyse polymorphisms among individual proteins and heterosis for agronomic traits in different parts of plants (leaf, roots, embryo and seeds) [49,50,54].

Analysis of the proteomic and transcriptomic data obtained from nuclear subcellular organelles, mitochondria, embryos at their developmental stages, isozymes and histone modifications have provided viable scientific support that proteins and their expressions could be essential “biomarkers” for heterosis [55]. These biomarkers can possibly be used as functional tools to assess the “hybrid vigor prospective” at a very early stage of development, contributing significantly to the crop improvement research [55]. 

Preliminary transcriptome studies on various crop species have established that the expression of favorable genes is predominant in hybrid plants compared to the parental inbred lines [27,50,56,57]. Modifications in gene expression patterns on a genome level and their respective action mechanisms in inbred lines and hybrids were documented in many plant species such as Arabidopsis [38,58,59], wheat [10], cotton [60], maize [56,61,62] and rice [37].

Transcriptome studies conducted at the translational phase of gene expression to estimate the relative contribution of each allele in a hybrid combination is considered as a potential approach for understanding the gaps in our knowledge of heterosis [18]. Comparison of gene expression levels has also been used to demonstrate that the interaction between parental genomes can result in modification of transcripts and protein abundance in the hybrid plants [21]. Steady changes in the protein expression at the transcriptional levels, along with additive and non-additive proteomic patterns have been observed in hybrids of several species [29,50]. For instance, studies on embryo, root and leaves have shown non-additive gene effects in differentially expressed proteins (DEPs) that determine hybrid vigor of various crops such as maize, wheat and rice [29,51,62]. These DEPs are found to be involved in signal transduction pathways, resistance mechanisms, photosynthesis and cellular metabolism, indicating the varying degree of heterosis and its dependency on these processes [63,64]. Genome-wide alterations in protein expression could help to achieve an inclusive understanding of heterosis [8]. However, to get a better apprehension, it is vital to consider the post-transcriptional and translational regulation of target alleles as studying the differential gene expression alone may not be enough to measure actual protein activity [1,50]. Post-translational modifications (PTMs) are also considered as determinants for heterosis; they are found to be critical in the regulation of proteins and their proper function [49]. 

### 2.5. Intrinsic Biological Processes Contributing to Heterosis

Plants are considered biological engines producing biomass with light energy and inorganic compounds as system inputs. In theory, this biomass is the differential of the energy going into the system and the energy consumed by the system [27]. Giving more energy or consuming less energy for metabolic processes of plants could result in increased plant growth and biomass accumulation. Therefore, the variation between two components of this equation provides the prediction of the phenotypic performance of a hybrid individual. This idea was proposed as a model describing the hybrids as more efficient than the inbred parents in terms of growth and energy consumption as they save a significant amount of energy from protein metabolism [65]. Such a fine-tuning of hybrids on a bio-energetic level made them capable of conserving energy that can be invested in increased growth rate and biomass production. In addition, Ni and colleagues proposed that resetting the circadian clock of plant species during daytime to a higher amplitude may lead to an increase in photosynthetic rate, and in turn, result in vigorous growth and biomass accumulation. Considering the energy dynamics of plants, it is obvious that a directional modulation of energy can possibly get translated into plant vigor and biomass [66]. This might be a reason that various hybrid combinations may achieve such a positive bio-energetic modulation during their whole lifespan or at certain developmental stages. 

Plant metabolomics on the other hand refers to the systematic identification and quantification of the plant metabolites (low molecular weight bio-chemicals) and to understand their role in systemic biology of plants [67]. It has been speculated that hybrids when compared to the parental inbred lines, exhibit a lower metabolic rate, resulting in a higher energy remainder for vegetative growth and biomass production [65]. A genetic explanation of such a scenario may correspond to the lack of allelic choice in homozygous state for an inbred line. Whereas, hybrids can have more alleles (especially in case of polyploid species), resulting in a robust metabolic profile as well as growth rate due to enhanced cell divisions [53]. Metabolome of a plant species serves as a bridge between the genome and phenome and provides a tool for understanding the complex connections between plant traits [68,69,70]. So far, little importance has been given to study plant metabolic profiles to understand the underlying phenomena of hybrid vigor. A recent study demonstrated that metabolites associated with yield from rice breeding lines grown in different conditions have shown an up-regulation of galactose metabolism that possibly promoted the heterotic effect [70]. A little significance of heterosis on metabolite level was also reported in a study conducted on interspecific introgression lines of tomato [68]. This study indicated that almost 50% of the mapped metabolic loci seems to be associated with the overall yield of tomato plants. Metabolic profiling of hybrid plants and using such data for genome-wide association studies would serve as an important tool for understanding the role of metabolites in the expression of hybrid vigor. 

One of the most recent development based on biological process model to explain heterosis traces back to the publication of Sewall Wright [71], who proposed that the metabolic flux of the hybrid *Aa* is higher than the mean parental metabolic flux due to the intrinsic non-linearity of the biological processes [71]. It remains one of the essential models in analyzing the superiority of hybrids regarding mongenic traits. Recently, the model is proven to be applicable to polygenic traits thanks to Rosas et al. [72], who accessed the flower asymmetry in *Antirrhinum,* and Fiévet et al. [73], who experimented the glycolysis/fermentation network in yeast [72,73]. Interestingly, Vasseur et al. [74] demonstrated the importance of Wright’s model in crops by testing growth rate and fruit number of *A*. *thaliana* and found out the allometric relationships between traits, occuring in both hybrids and parental lines, constrain phenotypic variation in a non-linear manner. More interestingly, these allometric relationships behave in a predictable pattern and could explain up to 75% of heterosis amplitude [74]. 

### 2.6. Mitochondrial Inheritance and Heterosis

**Figure 5:**
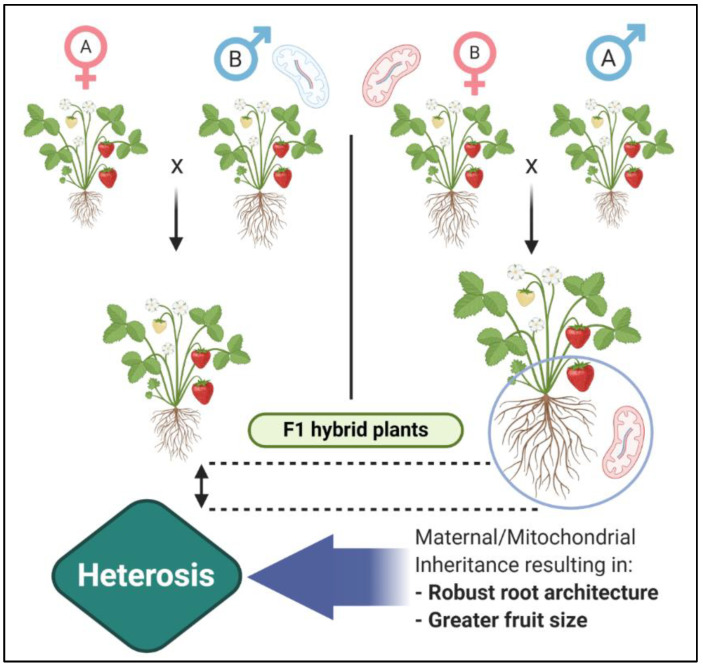
Hybrid vigor and maternal (mitochondrial) inheritance.

Apart from various biochemical, molecular, and physiological basis of heterosis, another significant aspect that caught the attention of plant scientists was the contribution of maternal inheritance in the manifestation of heterosis. Involvement of maternal inheritance in the phenomenon of heterosis has been suspected a long time ago when reciprocal crosses were observed to have variability in expression levels, demonstrating superiority of either of the resultant hybrids, owing to the specifically dominant role of the female parent [75]. An important attribute observed initially was higher respiratory rate during F1 hybrid germination that is related to mitochondrial respiration [76]. McDaniel, and Sarkissian [77] were the pioneers of this concept as they suggested a strong connection between mitochondrial complementation and heterosis in corn hybrids through their experiments for evaluation of differential efficiencies of mitochondria in terms of oxidative phosphorylation which demonstrated more efficient ATP synthesis in mitochondria of hybrids than that of inbreds and non-heterotic hybrids [75]. By comparing respiratory efficiency of mixtures of mitochondria from parental hybrids, an elevated synergic effect was observed which was, surprisingly, even more efficient than hybrids. Continuing validation of these findings through density gradient separation, presence of a distinct population of mitochondria was observed in a corn hybrid produced de novo rather than coming from either of the parents. Enzyme analysis showed elevated cytochrome c oxidase in hybrid and complementation effect in parental mixtures. With the same technique, multiple isozymes of malate dehydrogenase were detected in two mitochondrial populations of barley which were contrastingly different from their parents [75]. At that time, researchers were unable to comprehend the actual underlying mechanism but suspected that it might have arisen by an amalgamation of parental mitochondria or under the action of some genes or probably due to reasons unknown to scientists at that time [78]. But now, it is well known that mitochondrial DNA is maternally inherited and the mitochondrial heterosis can be explained in a better way. Figure 5 describes a simplistic way of mitochondrial inheritance of traits referring to the observed heterotic effect.

## 3. Heterosis in Self-Pollinated and Apomictic Plant Species

Modern molecular genetics suggest that dominance theory can be considered as a viable contributor to heterosis in many crops. In fact, a better understanding of the hyrbid vigor may need several genetic and epistatic factors to be taken into account as well [79,80]. Some primitive deductions on hybrid vigor were made on vegetable crops [81]. Soon after the availability of seeds by various industries, farmers opted for hybrid cultivars [80]. However, hybrid cultivars are more utilized in self-pollinated vegetable species compared to the cross-pollinated species [81]. Extreme hybrid vigor has been reported in some self-pollinated crops such as *Solanum melongena* L., *Capsicum annuum* L., *Solanum lycopersicum*, and *Lactuca sativa* which expressed higher heterosis (33–97%) than the parents [82]. The success of hybrid breeding also appears in bulb and root crops, thanks to the discovery and utilization of cytoplasmic male sterility (CMS). CMS was initially applied on *Allium cepa*, resulting in 14–67% increased yield compared to open-pollinated cultivars [81]. This achievement revolutionized the onion production industry and the hybrid breeding technique is now widely used for many root and bulb crops [81]. The possible reasons behind this self-pollinated heterotic phenomenon is a result of remarkable flower structures and a low out-crossing rate in onion [81]. A previous study about heterosis reveals that this phenomenon is simply the reclamation of inbreeding depression produced by essential genes [80]. However, in terms of quantitative genetics, hybrid vigor may possibly occur whenever there is genetic divergence among the parents that can also be apparent in self-pollinated species per se [81,83]. 

Note that although heterotic hybrids are well-known for exhibiting greater biomass, promptness of development, viable fertility and uniform progenies [20,80], these desirable traits can also be found in self-pollinated species. More interestingly, inbreeding depression is found to be higher in cross-pollinated crops in contrast to its self-pollinated counterparts [81]. Self-pollinated crops are extremely tolerant to inbreeding essentially with respect to environmental endurance. Such variety of performance by crops is believed to be associated with genetic balance. Reduced fitness in cross pollinated crop species is an outcome of heterozygous balance. Whereas, self-pollinated species display a potent homozygous balance leading to an overall progeny fitness that surpasses that of the heterozygous species [81].

In case of apomictic plant species, the phenomena of heterosis is not well-studied, even though apomixis offers a great deal of opportunities to exploit hybrid vigour. Genetic improvement in such plant species however is mainly based on traditional selection from natural ecotypes [84]. The convincing reason to focus on heterosis in apomictic plants owes to the fact that traits in apomictic hybrids remain fixed over generations and the maintenance of parental lines is unnecessary [85]. In addition to these advantages, the F1 hybrid seed in apomictic plants can be directly multiplied for advanced studies since the need of developing parental inbred lines is not a requirement per se, hence, it improves the efficiency and possibly speed-up the release of new cultivars as well [86]. Therefore, the exploration and utilization of the phenomenon of heterosis in apomictic plant species is an attractive goal for the plant breeding research community.

## 4. Future Perspective on Understanding and Utilizing Heterosis

Heterosis is a genome-wide phenomenon, which reflects global changes at both expression levels of genes and proteins [49], whereas existing models based on classical genetics are still popular among plant breeders [16]. The hybrid vigour for diploid genomes is explained quite confidently with dominance model, but in the case of polyploid species, it needs to be considered within the context of genome dynamics (cis, trans, and chromatin/epigenomic interactions) [16]. It has been suggested that hybrid vigor and the impact of self-pollination on this phenomenon can be better understood by using molecular markers, allozymes and sequencing plant genomes [87]. Using modern methods can result in breakthroughs and development of several supportive theories that can suggest factors other than heterozygosity, for example genetic diversity levels and causes of variances contributing to hybrid vigor [87]. 

**Figure 6:**
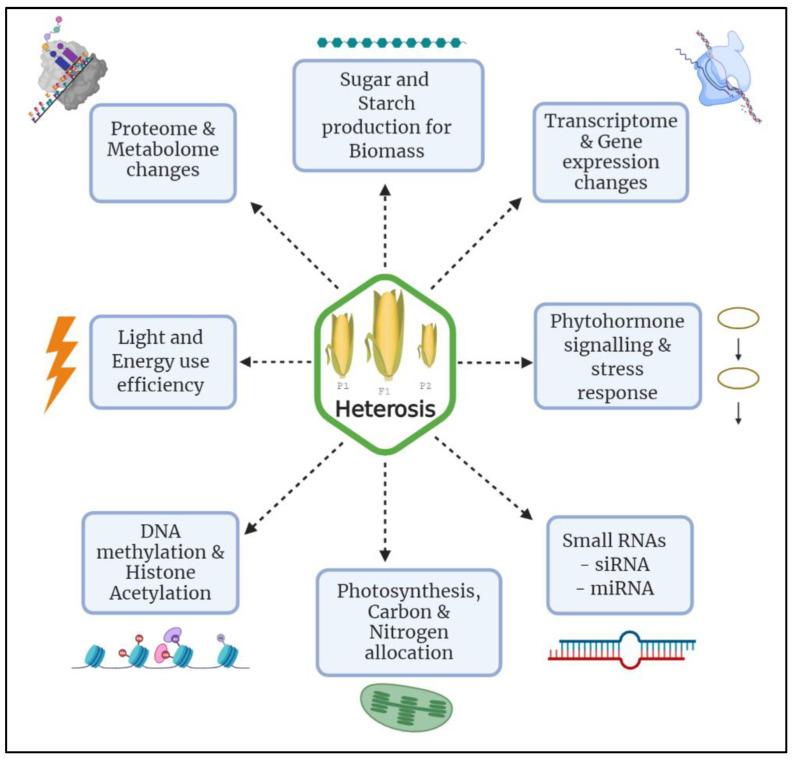
Different physiological and molecular mechanisms involved in manifestation of hybrid vigor.

According to the most sought-after dominance model of heterosis, superior alleles exceed the effect of the recessive alleles leading to the phenomena known as hybrid vigor and is by virtue of genetic divergence [82]. However, heterozygosity cannot be considered as the only fundamental contributor for the phenomena that results in an increased crop yield, fertility, and weight [79,88]. On the other hand, epigenetic effects [21], masking of deleterious alleles, dosage- sensitive genes, additive loci and overdominance [89] creates a barrier to understand the genuine reason for heterosis [90]. Therefore, heterosis in plants remains a topic requiring keen scientific investigations to develop a deep understanding and manifestation of the phenomena. Fortunately, the advent of molecular technologies has given us a hope to study heterosis to a great extent by various genome editing techniques (TALEN, CRISPR/Cas9) and molecular markers (RAPDs, SNPs) along with morphological and quantitative data [80]. Similarly, the biochemical tools continue to develop, and the research on hybrid vigor with respect to the contribution of proteome is becoming extensively detailed and specific. Most studies about the variations in protein structure and expression in heterosis mainly focus on a single developmental stage, whereas, proteins are found to be rich and vigorous in several developmental stages of crops, hence is a matter of further research and development. Similarly, genome-wide technologies and transcriptome level analyses also contribute significantly to the understanding of heterosis [50,91]. Applying models that account for multi-locus epistasis and using molecular tools to finely dissect genomic regions contributing to heterosis will allow a plethora of new insights to plant scientists. Figure 6. shows various physiological processes and molecular mechanisms involved in the manifestation of heterosis. Therefore, studies focused on these different aspects of plant biology has and will certainly help unravel the underlying basis of hybrid vigor. 

Apart from understanding the molecular mechanisms behind heterotic effect, it is often even more critical to utilize it in an economically efficient way. The knowledge obtained from the exploration of the underlying basis of heterosis can be applied in crop breeding, genetic improvement of parents, and development of superior performing hybrids by optimizing breeding schemes in various crops and ornamental species. In case of conventional hybrid breeding, the usual way is to screen a large set of individuals obtained through different crosses for traits of breeder’s intertest. Whereas, a very small number of tested individuals pass on to elite hybrid varieties after years of field testing. However, if the loci contributing to mechanism that result in heterosis and the causative variation are known, it becomes possible to narrow down the potential hybrid combinations based on strong predicted heterotic potential extracted from genomic information. Once after such a genotype-based prediction models reach higher accuracies, the time and labor cost to develop the hybrid variety can be reduced, hence increasing desired genetic gains. An effective strategy to increase the accuracy of these predictions may involve the utilization of large-scale well-tailored genomic data relevant to heterotic loci coupled with deep learning computational techniques [92]. Moreover, genomic selection can be one possible way to potentially decrease the cost of a hybrid breeding program by enabling the establishment of heterotic pools and decreasing the loss of genetic variation. An informative review on this topic has been recently published by Labroo and colleagues [93].

In case of self-pollinated crop species, the artificial emasculation is labor intensive and time consuming so the reliance on the development of male sterility becomes obvious. Acquiring and utilizing the knowledge of genes and molecular mechanisms involved in CMS could help coping with this bottleneck and opening new possibilities of hybrid breeding in crops where this has not been practiced previously. However, many practical issues still exist to exploit heterosis including utilization of the genetic diversity among parental lines, and accumulation of negative loci in F1 generation [1]. Similarly, wide hybridization might result in chromosomal aberrations and activation of transposons [94], leaving open challenges for plant scientists to explore viable use of genetic admixtures.

Taking a step further, speculations were already been made that by virtue of more knowledge and understanding of heterosis, it might be possible in the future to develop inbred lines with performance close to elite hybrids but without fulfilling the need of crossing among individuals [92]. We agree that this could be possible but will be very challenging to achieve due to the complexity of the genetic and molecular basis of heterosis. Nonetheless, further research is needed to not only understand the enigmas of hybrid vigor but to develop novel strategies to handle complex interactions and manage breeding platforms to meet the needs of global food security.

## Figures and Tables

**Table 1:** List of genes and their expression associated with various genetic traits contributing to heterotic effect in different plant species.

Gene	Crop/Plant	Arabidopsis Orthologue	Expression State	Impact	Reference
*ZAR1* (*Zea mays* ARGOS1)	Maize	ARGOS (Auxin Regulated Gene involved in Organ Size)	Overexpression	Increased organ size Increased Yield Improved drought tolerance	[29]
*CNR1* (Cell Number Regulator 1)	Maize		Silencing	Increased plant size Increased organ size	[29]
*SFT* (SINGLE FLOWER TRUSS)	Tomato	FT (FLOWERING LOCUS T)	Loss-of-function mutation	Enhanced yield	[28]
*AP2/EREBP* (APETALA 2/ethylene responsive element binding protein)	Arabidopsis		Over-expression	Cell proliferation Enhanced heterotic effect	[30]
*CCA1* (CIRCADIAN CLOCK ASSOCIATED 1)	Arabidopsis		Epigenetic modification	Increased vigor in plant development Increased biomass production	[8]
*LHY* (LATE ELONGATED HYPOCOTYL)	Arabidopsis		Epigenetic modification	Increased vigour in plant development Increased biomass production	[8]

Abbreviation for Gene names are capital letters and italicized, the respective full-forms are mentioned in parenthesis ().

## Data Availability

Not applicable.
